# Smart green spectrophotometric estimation and content uniformity testing of chlorphenoxamine HCl and caffeine in bulk forms and combined pharmaceutical formulation

**DOI:** 10.1038/s41598-025-92166-7

**Published:** 2025-03-19

**Authors:** Ahmed Ashraf, Ghada A. Sedik, Badr A. El-Zeany, Yasmin Rostom

**Affiliations:** Analytical Chemistry Department, Faculty of Pharmacy, Cairo University, Kasr El-Aini Street, Cairo, 11562 Egypt

**Keywords:** Chlorphenoxamine HCl, Caffeine, Content uniformity test, Factorized response spectrum, Whiteness assessment, Medical research, Chemistry, Physics

## Abstract

Chlorphenoxamine hydrochloride is a chemical that has attracted interest because of its notable anti-histaminic and anti-cholinergic characteristics. Moreover, it has been recognized as a highly efficient tool in the fight against several lethal viral diseases, such as Severe Acute Respiratory Syndrome Coronavirus. In this study, five efficient and straightforward univariate spectrophotometric approaches are proposed for accurately measuring the quantities of Caffeine and Chlorphenoxamine HCl in bulk forms and combined pharmaceutical formulations. Notably, these methods based on an advanced approach using the factorized response spectrum and not require any initial processing.They have been classified into three spectrophotometric platform windows. The study of Window I focuses on absorption spectra of substances in their original states(zero-order). It includes, absorbance resolution method (AR), extended absorbance difference method (EAD), and factorized zero order method (FZM). Window II focuses on the factorized derivative method (FDM), while Window III focuses on factorized ratio difference method (FRM). These approaches successfully measured concentration of Caffeine and Chlorphenoxamine HCl within a range of 3–35.0 and 3–45.0 μg/mL, respectively. The factorized response spectrum’s exclusivity stems from its capacity to fully separate the mentioned components in mixture and recover the pure spectra. Validation of the suggested approaches has been conducted according to guidelines established by International Council for Harmonization, which demonstrated acceptable levels of accuracy and precision. The scope of this work has been expanded to include verification of content uniformity of dosage units according to recommendations outlined in United States Pharmacopoeia. Greenness profile of the proposed approaches has been properly assessed using state-of-the-art software metrics, in comparison to the reported one. Finally, the proposed methods demonstrated strong compliance with the recently established principles of white field of analytical chemistry.

## Introduction

### Overview

**Fig. 1:**
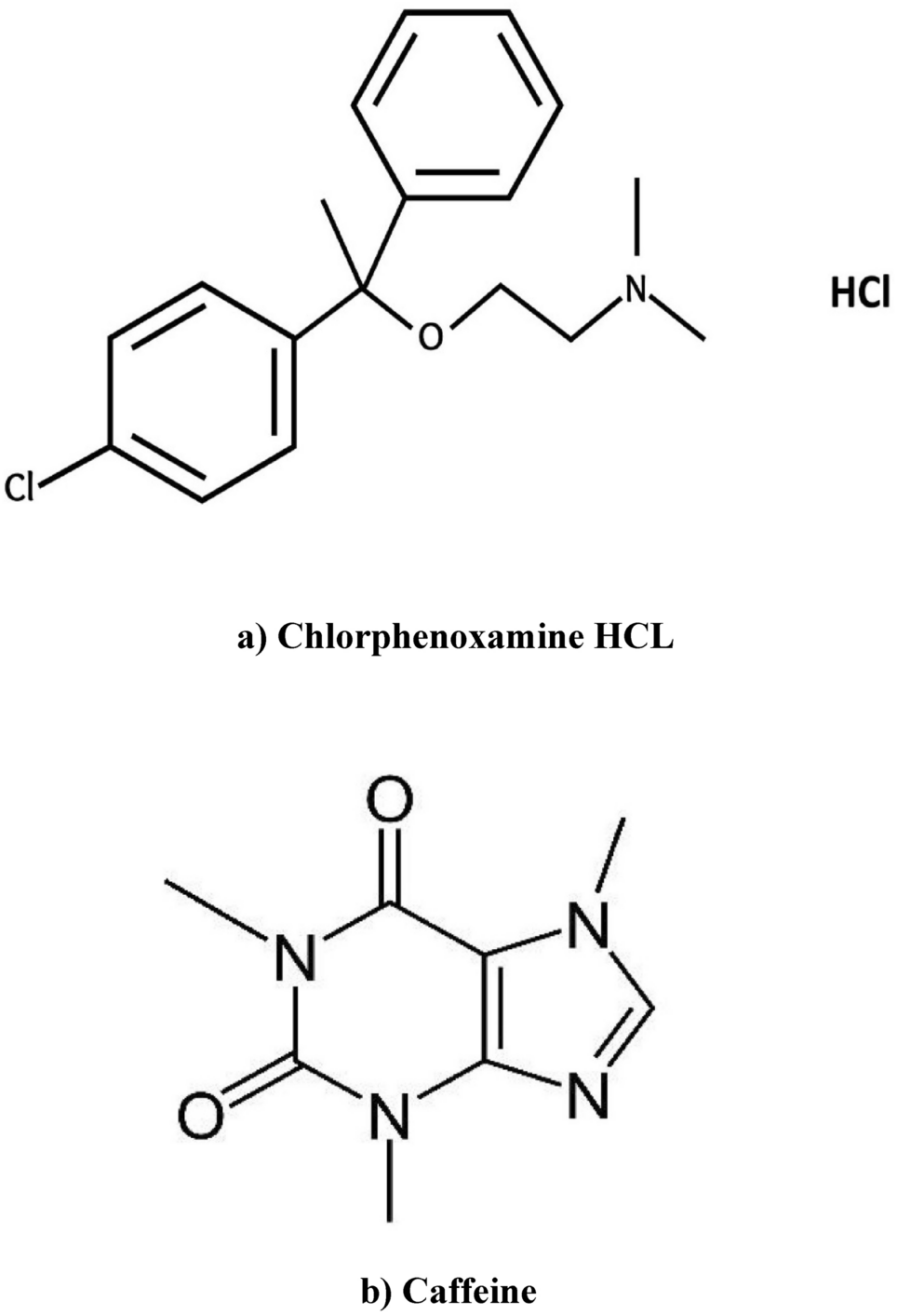
Chemical Structure of (**a**) Chlorphenoxamine HCl, (**b**) Caffeine.

The application of FDA-approved pharmaceuticals for off-label use is considered a more cost-effective and safer approach than developing novel therapies for the treatment of infectious diseases. The use of reprocessed prospective pharmaceuticals offers advantage of picking selecting compounds that have passed clinical investigation, involving research on pharmacokinetic properties along with safety information. Retained seek for efficient and conveniently available protective or alternative treatments for serious viral diseases continues, despite potential benefits of repurposed medications with proven safety profiles^1^0.0Chlorphenoxamine0hydrochloride0(CPX);2-[1-(4-chlorophenyl)-1-phenyl ethoxy] -N, N-dimethyl ethanamine, Fig. 1a, is a compound that has garnered interest due to its notable anti-histaminic and anti-cholinergic effects^2^. CPX possesses a characteristic that renders it highly advantageous in treatment of ailments like hay fever, rhinitis due to allergies, and dermatitis. CPX has demonstrated efficacy in treating many allergy-related disorders that are linked to cholinergic activity, in addition to its anti-histaminic effects^3^. CPX has shown promise in treating multiple lethal viral diseases, such as SARS-CoV, MERS-CoV, and EBOV. This is mainly attributed to its exceptional qualities as a G Protein-coupled receptor antagonist, which is further demonstrated by FDA-approved drugs for off-label purposes^1,4,5^. Caffeine (CAF); 1,3,7-trimethylpurine-2,6-dione^6^, Fig. 1b, is consistently paired with CPX due to its ability to counteract the sedative effects of CPX, thereby enhancing its central stimulant properties^7^. Different analytical techniques, such as HPLC^8,9^, TLC-densitometry^10^, Spectroscopy^11–13^, Voltammetry^14^, and potentiometric method^15,16^, were published for CPX analysis in pharmaceutical products.

Spectrophotometry approach has become more important due to its various uses, such as substance recognition, reaction observing, and quantification objectives^17–22^. Furthermore, it provides comparable dependability and precision as diverse chromatographic methods, without requiring sophisticated computer systems, extensively trained analysts, or laborious troubleshooting procedures^23–25^. However, the use of combined chromatographic devices is both tedious and economically costly due to the large consumption of expensive organic solvents, in addition to the substantial costs associated with chromatographic columns along with replacement components^23,24^. Spectrophotometry has received significant focus as a viable option that is both time and cost efficient, as well as environmentally benign. It involves the use of less harmful solvents and instrumental energy, resulting in less waste production^26–28^.

### Work objective

Multiple spectrophotometric approaches without separation have been documented for simultaneous determination of drug combinations with overlapping spectra, demonstrating their reliability. These methods relied on basic findings accompanied by simple data manipulation for distinguishing overlapping UV-spectra. Nevertheless, traditional UV-approaches require difficult manipulation processes, the use of specialized software that must be purchased, and strict prerequisites that hinder their simple implementation. In this study, the incorporation of the factorized response spectrum (FRS) into the response data has been determined as a critical method in spectrophotometric approach. It enhanced the selectivity of the technique, making it comparable to chromatographic methods. Additionally, the FRS method required less solvent, had lower costs, and required minimal effort. Subsequently, the scope of the research was expanded to guarantee the consistency of the drug in each dosage unit, which is a necessary need prior to undertaking bioequivalence tests^29^. the manipulation of the analytes’ spectra can be carried out using the integrated software of any spectrophotometer now accessible, without any impact on the outcomes. The potential effects on environment, health, and safety of the suggested approaches have been evaluated using credible and current technologies like analytical Eco-Scale^30^, Complex GAPI^31,32^, and AGREE software^33^. RGB-12 algorithm excel sheet was used to promote the harmonious integration of analytical, ecological, and practical aspects of the proposed approaches^22,28,34^. Our aim is to provide a clear understanding of environmental impacts of our methodology compared to method described in the reference. Established procedures were validated in accordance with the ICH standards^35^.

### Theoretical background for the advanced factorized response techniques

In the current research, determination of each component in the target binary combination was achieved using various advanced resolution strategies applying factorized response spectrum (FRS). Five approaches rely on using zero /or derivative /or ratio spectra without the need for preliminary separation steps and do not need a search for zero-crossing points. A response value is calculated for component Y in the mixture where X has no effect or cancelled, then multiplied by the equivalent FRS of Y utilizing the similar manipulated mode (D^0^ or D^n^) to extract Y spectrum at its corresponding mode. While the second component (X) can be obtained at its corresponding mode via spectrum subtraction method.

#### Absorbance resolution method (AR)

AR is suitable for analyzing drugs spectra at two distinct wavelengths^36–38^. Y component has a significant difference in absorbance values, while X component shows the similar absorbance at the certain wavelengths. Consequently, X component can be disregarded using dual wavelength method. Factorized response spectrum (FR_ΔA_S) showing a_Y_/[Δa_Y_]′ is obtained from division of zero order spectrum (D^0^) of one original concentration of Y within linearity range by the absorbance difference values at two selected wavelengths where exclusion of X will be accomplished.

$${\text{FR}}_{{{\Delta A}}} {\text{S of Y = a}}_{{\text{Y}}} {/ }\left[ {{\Delta }\left( {{\text{a}}_{{1}} {\text{{-} a}}_{{2}} } \right)} \right]^{\prime}$$

Determining D^0^ spectrum of Y in corresponding binary mixture, absorbance difference (ΔA) value at specified dual wavelength is first manipulated then multiplied by FR_ΔA_S of Y.

#### Extended absorbance difference method (EAD)

The EAD technique is used to study the D^0^ spectra of a binary mixture where, the spectrum of interest component (X) exhibits a wider range compared to the interfering component (Y). The absorbance difference value (∆A) for the component Y is extended, indicating that it does not contribute much to the mixture^25,39^. The factorized spectrum of X is produced using software programs by division the concentration of the original D^0^ spectrum of X, as defined by Beer’s law, by the difference in absorbance between two chosen wavelengths in the expanded region where the other component does not have an effect.

$$\text{FRS of X}= \frac{X (D^\circ ) }{\Delta A}$$

The goal of EAD is to obtain the original D^0^ spectrum of the X component by multiplying the extended absorbance difference value of the determined lab mixture with the factorized extended absorbance difference spectrum of the subjected drug.

#### Factorized zero order method (FZM)

A single response value is determined for each target analyte, excluding any effect from the other components of the mixture, by utilizing zero-order, derivative, and ratio spectra^18,40^.

Factorized spectrum via absorbance value is derived by dividing D^0^ spectrum of any concentration pure X by its absorbance value at specified wavelength λ_S_ (maxima or isopoint)^41^.

- Factorized spectrum via absorbance value= a_X_/ [a_X(λs)_]^’^

D^0^ spectrum of X in the binary mixture is determined by multiplication absorbance value at λmax, whether it has been captured or estimated, by its corresponding factorized spectrum.

#### Factorized derivative method (FDM)

First order derivative spectra (D^1^) have been attained by calculating the derivative of the absorbance spectra of the prescribed drugs using a wavelength difference (Δλ) of 4 and a scaling factor of 10. A direct relationship has been established between the amplitude of the first derivative spectra and the measured concentrations. Factorized amplitude spectrum is derived by division the derivative spectrum (D^n^) of the original X concentration by its stored amplitude value at given wavelength (λs), which can be either a maximum or a minimum value (without any contribution from other analytes).

- Factorized amplitude spectrum = [d/d_λ_ a_X_]/ [d/d_λ_ a_X_]_( λS)_

The derivative spectrum (D^n^) of X is calculated by multiplication of the amplitude value of the mixture at λs by its equivalent factorized amplitude spectrum. Performing spectral subtraction of X from corresponding mixture, Y spectrum restored.

Derivative transformation is employed to extract D^0^ spectra from D^n^ via multiply the derivative spectra of each analyte by the corresponding decoding spectrum derived by division normalized spectrum of each component by its equivalent derivative^19^.

#### Factorized ratio difference method (FRM)

FRM is applied for determining a complete spectral overlap binary mixture (X and Y)^40^. Ratio spectra of each drug separately were established by dividing them by the spectrum of X as the divisor. As a result, ratio spectra were obtained for X and Y. Ratio spectra of X are consistently alignment with the wavelength line. Subsequently, calibration graphs have been generated: Linearity was assessed by plotting the variation in amplitudes of ratio spectra against corresponding concentrations of Y and plotting constant values of X against its corresponding concentrations.

The factorized amplitude difference spectrum of pure Y is derived by dividing the ratio spectrum of any measured concentration of Y using the X" divisor by amplitude difference value at two specific wavelengths (λs).

- Factorized amplitude difference spectrum = [aY/[aX aX(λs) /$\Delta$aY(λs)]^’^

Two options are available: The initial approach entails acquiring the ratio spectrum of Y through the multiplication of its associated factorized amplitude difference spectrum with the amplitude difference value of Y present in the mixture. Spectrum subtraction approach extracts the ratio spectrum of X in the combination. This ratio spectrum remains constant across the whole spectrum^19,42^. D^0^ X spectrum is recovered by multiplying the extracted constant value by its divisor.

## Experimental

### Instruments and software

The experiment utilized a Shimadzu double beam UV–visible spectrophotometer (model 1800, Tokyo-Japan) that was programmed with UV Probe 2.43 software. A spectrophotometric examination has been performed utilizing a series of identical quartz cells, each with a length of path of 1 cm. The analysis covered a range of wavelengths of 200.0–400.0 nm with a 1 nm interval. All measurements have been performed. The recording speed is 2800 nm/min. Spectral band was finely adjusted to a width of 2 nm.

### Materials and chemicals

The solvent used was double distilled water. The CPX and CAF standards have been acquired from EIPICO Pharmaceuticals in 10th of Ramadan City, Egypt. The published technique indicates that the CPX standard has a purity of 100.06 ± 0.47% and the CAF standard has a purity of 99.43 ± 0.60%^8^. Allergex Caffeine tablets, manufactured by EIPICO Pharmaceuticals, were obtained from nearby pharmacies. Each tablet contains 20 mg of CPX and 50 mg of CAF.

### Standard solutions

Standard stock solutions (1.0 mg/mL) have been produced separately for each drug by double distilled water as the solvent. Further diluting of the initial stock solution has been carried out in order to achieve a concentration of 100.0 μg /mL for the CPX and CAF working solutions.

### Procedures

#### Spectral features

**Fig. 2:**
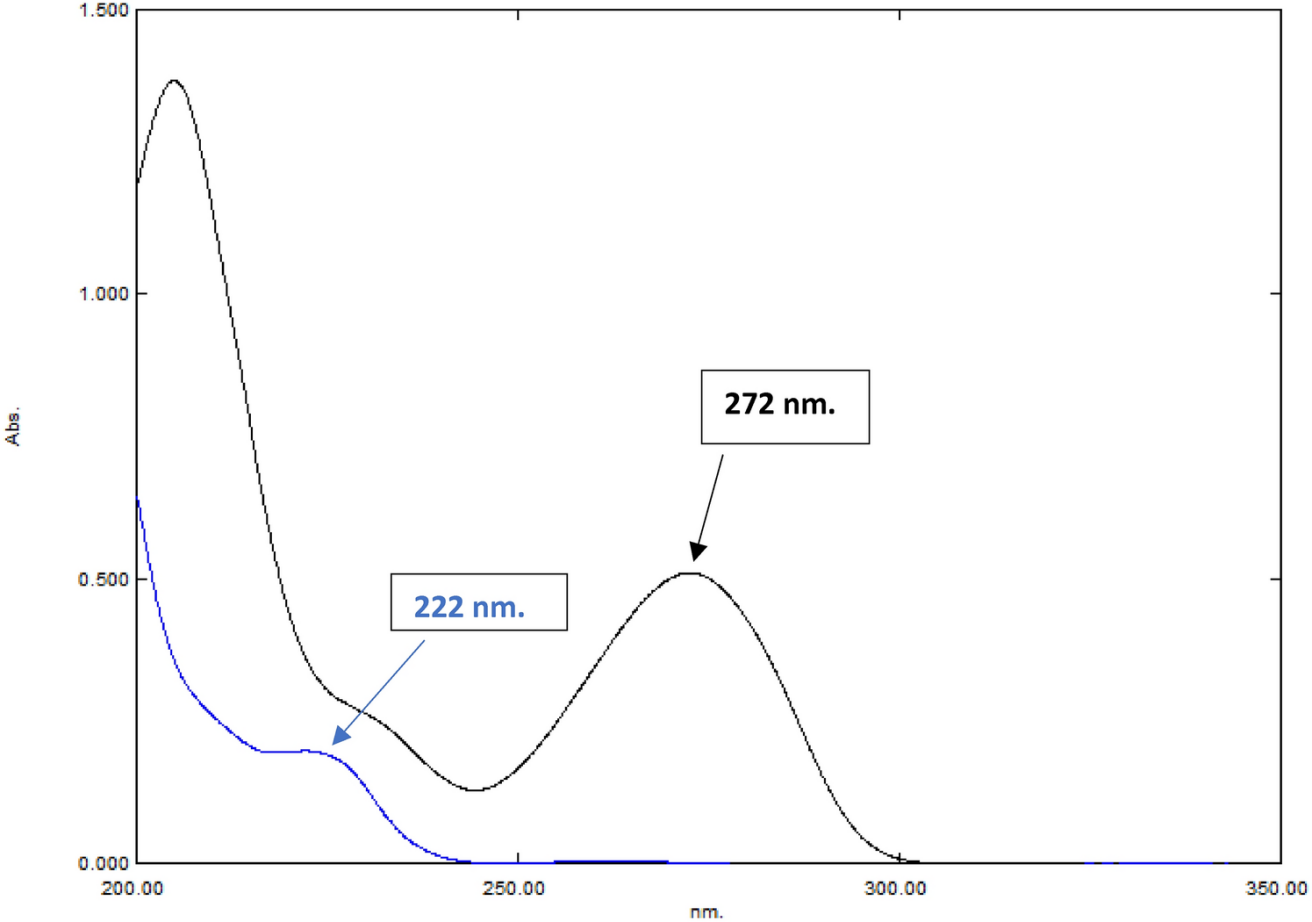
Zero order absorption spectra of 10.0 μg/mL CAF (**—**) and 4.0 μg/mL CPX (**—**).

Various standard solutions have been produced in double distilled water with concentrations ranging from 3.0 to 45.0 μg /mL for CPX and from 3.0 to 35.0 μg /mL for CAF. These solutions have been divided into two sets of 10-mL volumetric flasks. The solutions were partitioned into two sets, each contained in 10-mL volumetric flasks. Figure 2 presents the D^0^ spectra of each drug individually and in mixture, using a wavelength in the range of 200.0–400.0 nm. By comparing it to double distilled water, we observed that CAF exhibits maximum absorption at 272.0 nm, while CPX exhibits maximum absorption at 222.0 nm. This can be attributed to the higher absorptivity and greater sensitivity of these drugs. Subsequently, combinations generated in the laboratory with different ratios were created and compared to double distilled water as a reference. Absorption spectra obtained have been stored in the computer and subsequently subjected to extra processing interprets for each method as described below, section "Analysis of binary laboratory mixture".

#### Calibration curves construction

##### AR, EAD, FZM, FDM_b_, and FRM_b_ (Window I)

The maximum absorbance spectra of D^0^ at 272.0 and 222.0 nm have been measured for the concentrations of CAF and CPX. A regression equation was then obtained based on these values.

##### FDMa (Window II)

The D1 derivative spectra were acquired by performing differential analysis on absorbance spectra of mentioned drugs using a wavelength in the range of Δλ = 4 and a scaling factor of 10. A linear relationship was established among amplitude of the first derivative spectra at 287 and 231 nm and concentrations of CAF and CPX, respectively.

##### FRMa (Window III)

The D^0^ spectra of CAF and CPX were split individually by the spectrum of 10.0 μg/mL of CAF as a divisor. As a result, ratio spectra have been obtained for CAF and CPX. The ratio spectra of CAF remained constant along the wavelength axis. Subsequently, successive calibration graphs were conducted: The relationship between the difference in amplitudes at 235 and 225 nm in the ratio spectra and the concentrations of CPX shows linearity. Similarly, calculated constant values obtained for CAF also show linearity with its concentrations.

#### Analysis of binary laboratory mixture

Different solutions with Various ratios of CAF and CPX were obtained in 10-mL volumetric flasks to assess specificity of the suggested approaches. The spectra obtained were recorded using a wavelength in the range of 200.0—400.0 nm.

### Application to pharmaceutical formulation

Ten tablets have been finely powdered in a mortar. Each tablet is labelled with the quantity of 20 mg of CPX and 50 mg of CAF. In a conical flask, a precise amount of substance, supposed to consist of 20 mg of CPX and 50 mg of CAF, has been then transferred using 20 mL of double distilled water and subjected to sonication for approximately 20 min. Filter paper was used to filter the contents of the conical flask and transfer them into a 50-mL volumetric flask. The remaining substance has been subsequently washed triple with double distilled water. This quantity has been diluted to a mark using the identical solvent, resulting in a final solution of 4.0 μg /mL for CPX and 10.0 μg /mL for CAF. Steps were subsequently performed according to the instructions provided in each approach.

### Content uniformity test

Similar extraction and preparation processes outlined in section "Application to pharmaceutical formulation" have been utilized to assess consistency of the content in the commercially available tablets. However, instead of employing multiple tablets, just one complete tablet was used for the evaluation. For each of the ten trials, a tablet was extracted using 20 mL of double distilled water. The extraction process involved sonication for approximately 20 min and filtering through filter paper into a volumetric flask with a capacity of 50 ml. The residue has been washed three times with double distilled water and then adjusted to the desired amount using the identical solvent. This resulted in a solution with a concentration of 4.0 μg /mL for CPX and 10.0 μg /mL for CAF. The suggested approaches have been utilized to determine concentration of CPX and CAF, and the evaluation of content uniformity has been subsequently accomplished according to the recommendations set by the USP^43^.

## Result and discussion

Spectrophotometry is a widely used and indispensable method in QC labs around the world for pharmaceutical analysis. Presence of overlapping spectra in a pharmacological mixture poses a challenge in directly determining their individual components. Therefore, an effective mathematical resolution technique is essential, unless mechanical separation is necessary. Furthermore, drug combinations that possess distinct spectral characteristics promote advancement of novel spectral resolution approaches that outperform established methods by reducing need for extensive manipulation and providing additional benefits. Additionally, spectrophotometry has made significant improvements in field of green analytical chemistry by substituting harmful solvents with eco-friendly alternatives. The study presents development of novel and environmentally friendly spectrophotometric techniques.

### Method optimization

#### Solvent selection

Development of a sustainable spectrophotometric method often begins with the careful selection of an optimal solvent. This solvent should possess several key characteristics, such as sufficient dissolution for all drugs tested, good signal sensitivity, and minimal background noise. However, it is important to consider the potential detrimental influence of the solvent on both human health and the environment. Distilled water was discovered to exhibit excellent solubility for both drugs and provide comparable absorption spectra. To enable analysis at lower wavelengths without any interference from the solvent, a solvent with an extremely low UV cut-off, such as water, has been recommended. According to the Green Chemistry, water is considered the most environmentally sustainable and cost-effective solvent in comparison to other solvents of organic origin^44^. Water was chosen as the solvent for this study due to its sustainability, affordability, widespread availability, non-toxic nature, and its ability to effectively dissolve the drugs being investigated.

#### Wavelength selection

Analyzing D^0^ spectra of each component individually and in mixture, using a wavelength in the range of 200.0–400.0 nm along with contrasting it to double distilled water, we found that CAF has a maximum absorption at 272.0 nm, whereas CPX has a maximum absorption at 222.0 nm, as shown in Fig. 2.

#### The factorized response spectrum investigation

**AR:** The factorized spectrum has been obtained by dividing the D^0^ spectrum of 5.0 μg/mL of CPX by the difference in absorbance values between 230 and 256 nm.

**EAD:** The factorized spectrum is obtained by division the D^0^ spectrum of CAF according to Beer’s law, utilizing the absorbance difference between two specified wavelengths (275 and 285) Within the expanded region, zero contribution is detected from additional components.

**FZM:** The factorized spectrum of CAF has been obtained by dividing the pure spectrum of a 10.0 μg/mL of CAF by its absorbance value at its maximum wavelength (λmax) of 272 nm.

**FDM:** The factorized amplitude spectrum of CAF has been obtained by dividing the D^1^ spectrum of a reference solution containing 10.0 μg/mL of CAF by its amplitude value at a wavelength of 287 nm.

**FRM:** The factorized amplitude difference spectrum of CPX has been derived by dividing the ratio spectrum of a standard CPX solution with a concentration of 5.0 μg/mL by the amplitude difference between 225 and 235 nm (P225 nm – P235 nm).

### Method development

In this current research, we have devised a significant method for spectral analysis by employing factorized response spectra of the analytes namely: AR, EAD, FZM, FDM, and FRM. These spectra are obtained in various conditions, such as zero-order, derivative, or ratio spectra. Implemented techniques were utilized to quantitatively assess the levels of CAF and CPX. A statistical comparison was conducted to assess the effectiveness of the various factorized response spectra in accurately determining target analytes.

### Advanced factorized response technique

The key characteristic of these approaches is their capability to separate the D^0^ absorption spectra of individual components from mixtures with overlapping spectra by obtaining a fixed numerical value^45^. As a result, the medications can be accurately measured at their sensitive λ_max_, Fig. 2, eliminating the need to capture for wavelengths with zero-contribution in derivative spectrophotometric approaches. The recovery of the D^0^ spectrum facilitates the verification of drug authenticity and purity in QC labs. This is because the D^0^ spectrum acts as a distinctive fingerprint to each component.

#### Absorbance resolution method (AR)

**Fig. 3:**
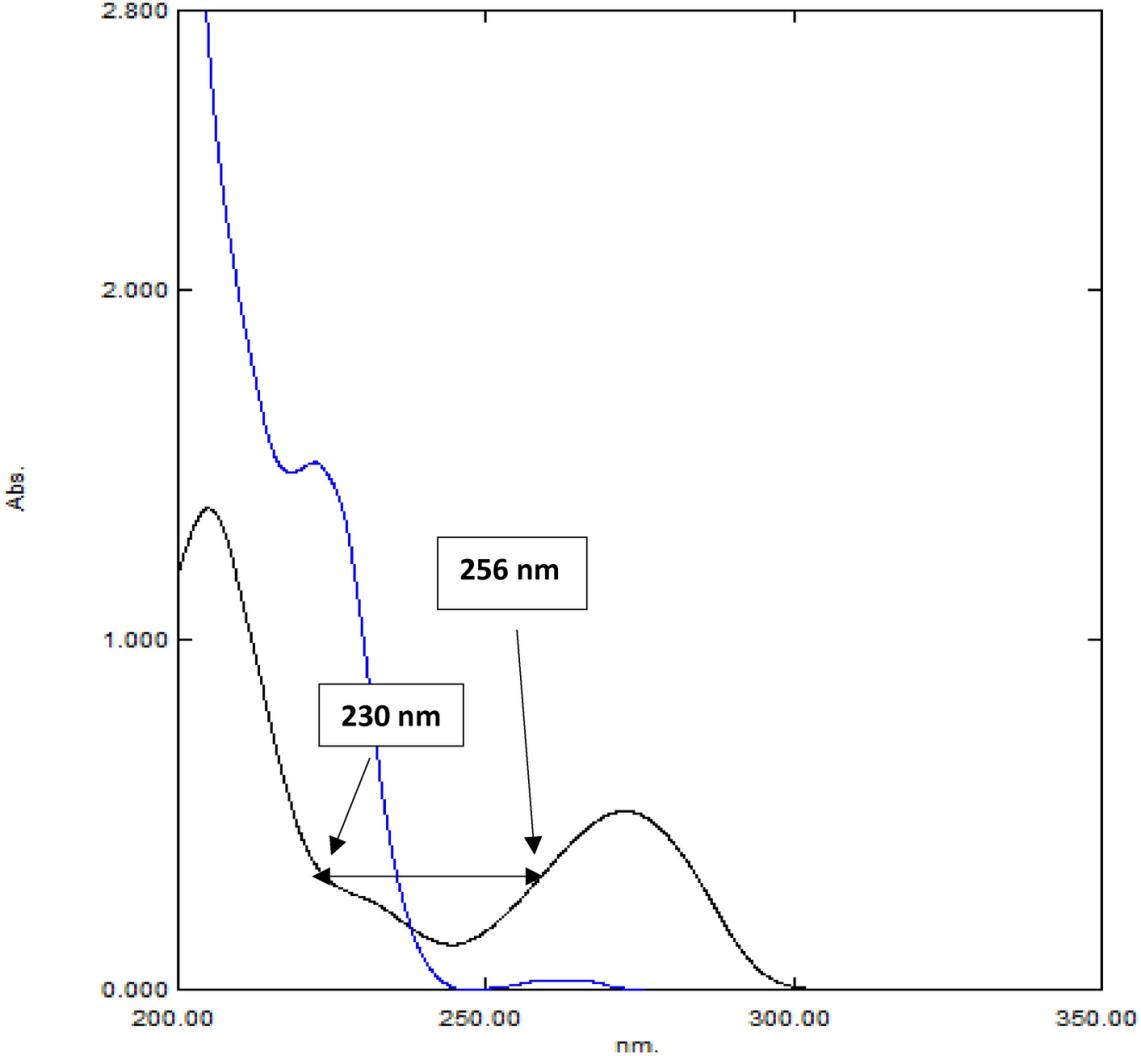
The zero-order spectrum (D^0^) of 10 μg/mL CAF (**—**) and 35 μg/mL CPX(**—**) at which two selected wavelengths (230 and 256 nm) showing constant absorbance of Caffeine.

The most important step involves creating the resolving tool FR_ΔA_S of CPX via dividing D^0^ absorption spectrum of 5.0 μg/mL CPX by a difference in absorbance values at specified wavelengths A_230nm_ and A_256nm_ during which CAF has the same absorption, Fig. 3. D^0^ spectra of CPX were obtained by multiplying generated FR_ΔA_S by absorbance difference (A_230nm_ − A_256nm_) in each Lab-Mix. D^0^ spectra of CAF, which were determined by subtracting resolved D^0^ spectra of CPX from that of corresponding Lab-Mix. Concentrations of each analyte in Lab-Mix was computed using the relative regression equations^36^.

#### Extended absorbance difference spectrophotometric method (EAD)

The ΔA value was determined by measuring difference in absorbance at the expanded wavelength range of 275 and 285.0 nm in the scanned D^0^ spectra of the prepared mixture. Result has been multiplied by the pre-calculated CAF factorized extended absorbance difference spectrum in order to obtain pure CAF^25,39^.

#### Factorized zero order method (FZM)

**Fig. 4:**
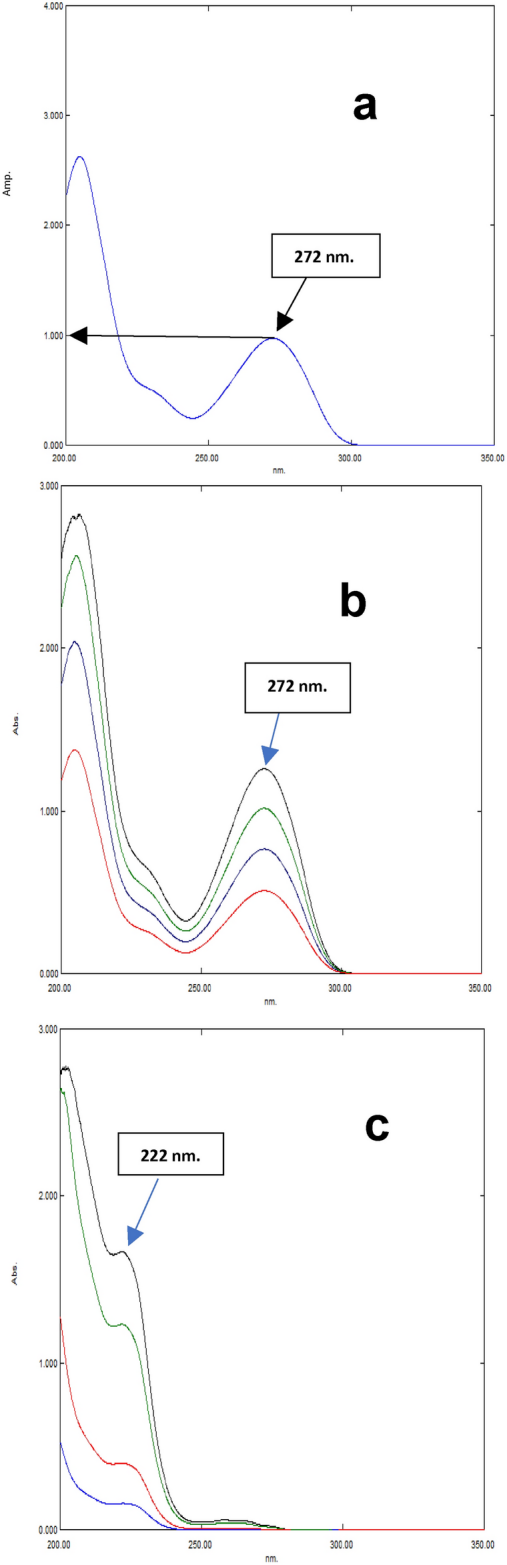
**(a)**- Factorized spectrum of the zero order of CAF (10.0 μg/mL), (**b**)- The zero order absorption spectra of CAF (10, 15, 20, 25 μg/mL) obtained after manipulation of the laboratory prepared mixtures, and (**c**)- The zero order absorption spectra of CPX (4, 10, 30, 40 μg/mL) obtained from subtraction of the spectra of CAF from those of the laboratory prepared mixtures.

CAF spectrum was factorized by dividing the D^0^ of a 10.0 μg/mL solution of CAF by its absorbance value at λ_max_ 272 nm, Fig. 4a. The measured absorbance readings of the laboratory-generated mixes were obtained at a wavelength of λ_max_ 272 nm. Subsequently, each individual value has been multiplied by the factorized spectrum of CAF, resulting in replication of the pure D^0^ of CAF across each combination^40^. The concentration of CAF in all combinations has been determined utilizing the following regression formula; A_272 nm_ = 0.0499 C_CAF_ + 0.0119, Fig. 4b, Then, D^0^ spectrum of each CAF sample was subtracted from that of matching laboratory-prepared mixture, resulting in the restoration of the original CPX spectrum in each mixture, Fig. 4b. The concentration of CPX in the mixes has been determined using the following regression formula: A_222nm_ = 0.041 C_CPX_ + 0.0155.

#### Factorized derivative method (FDM)

**Fig. 5:**
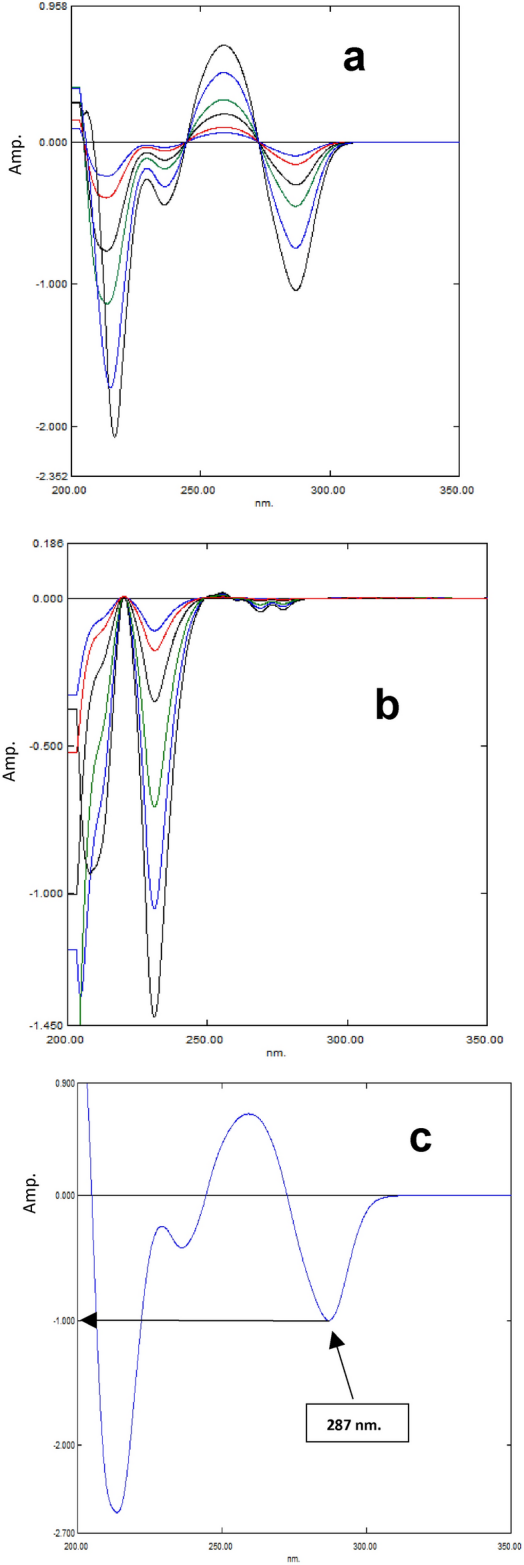
(**a**)- First derivative spectra of CAF (3,5, 10, 15, 25, 35 μg/mL), (**b**)- First derivative spectra of CPX (3, 5, 10, 20, 30, 45 μg/mL), and (**c**)- factorized CAF spectrum obtained by dividing the D^1^ spectrum of standard CAF (10.0 μg/mL) by its amplitude value at 287 nm. In this figure: Δλ = 4 and scaling factor = 10.

The D1 spectra of CAF and CPX were obtained by calculating the derivative of the absorbance spectra of the required components using a Δλ of 4 and a scaling factor of 10, Fig. 5a, and 5b. The amplitude spectrum of CAF has been factorized by dividing the D1 spectrum of a reference solution containing 10.0 μg/mL of CAF with amplitude value at 287 nm, as shown in Fig. 5c. Amplitude values of laboratory-generated mixes at 287 nm were recorded independently of CPX. The factorized spectrum of CAF was used to multiply each value, resulting in the accurate reproduction of the characteristic D1 of CAF in each combination. Determination of CAF concentration in each mixture has been accomplished using regression equation; P_287nm_ = 0.0296 C_CAF_ + 0.0073. Each D^1^ spectrum of CAF that was acquired has been subtracted from matching D^1^ spectrum of laboratory created mixture. CPX concentration in the mixes has been determined using regression equation: P_231nm_ = 0.0358 C_CPX_ + 0.0049. Derivative transformation is utilized to obtain zero-order absorption spectra from the derivative spectra of CAF and CPX, obtaining D^0^ of each drug is a straightforward process (FDM_b_). Simply the derivative spectra of CAF and CPX is multiplied by the decoding spectrum for each drug^19^. This decoding spectrum is attained by dividing normalized spectrum of each component by its equivalent derivative [a CAF]/(d/d_λ_) [a CAF] and [a CPX]/(d/d_λ_) [a CPX], respectively.

#### Factorized ratio difference method (FRM)

**Fig. 6:**
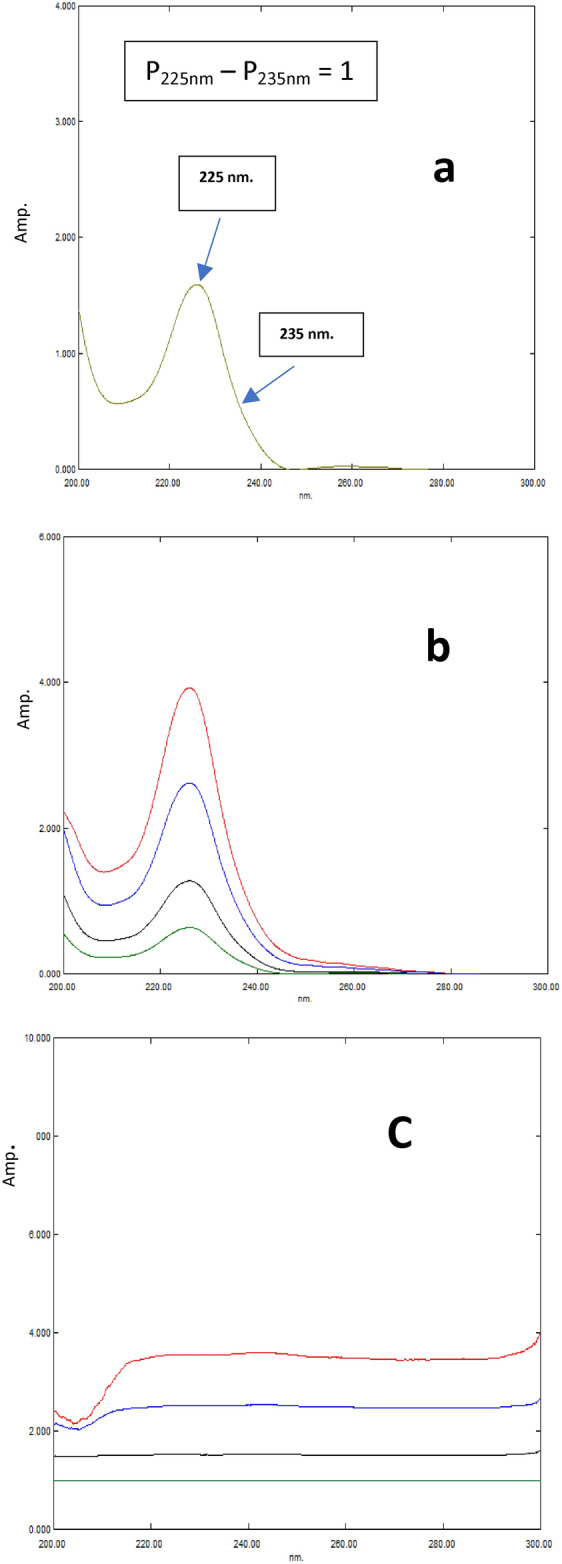
(**a**)- Factorized spectrum of CPX (5.0 μg/mL) obtained from the difference in amplitude between 235 and 225 nm in the ratio spectrum, (**b**)- The ratio spectra of CPX (4, 10, 30, 40 μg/mL) obtained after manipulation of the laboratory prepared mixtures, and (**c**)- The constants obtained from subtraction of ratio spectra of CPX from the corresponding laboratory prepared mixtures.

FRM method has been employed to generate whether ratio spectra FRM(a) or zero order absorption spectra FRM(b) for CPX and CAF. Ratio spectra of CPX have been calculated by dividing its D^0^ (3.0–45.0 μg/mL) by the standard 10.0 μg/mL CAF as a divisor. Two selected wavelengths, 235 and 225 nm, were determined to be appropriate due to a significant disparity in the CPX amplitude values at these wavelengths with respect to concentration. In contrast, ratio spectrum of CAF exhibited a consistent amplitude. Factorized amplitude difference spectrum of CPX has been obtained by dividing ratio spectrum of CPX (10.0 μg/mL) by amplitude difference between 235 and 225 nm (P235 nm – P225 nm), Fig. 6a. For maintaining the accuracy of factorized amplitude difference spectrum, it is important that the value of (P235 nm—P225 nm) is equal to 1. The laboratory-prepared mixes were analyzed by recording the amplitude values of their ratio spectra at various wavelengths. Subsequently, each individual value has been multiplied by factorized amplitude difference spectrum of CPX, resulting in reproduction of the pure ratio spectra of CPX, Fig. 6b. The ratio spectrum of each CPX sample has been subtracted from corresponding ratio spectrum of laboratory generated combination in order to obtain the constant values (CV), Fig. 6c, which reflect the CAF ratio spectra. Subsequently, these unchanging values were documented^40^. Additionally, the FRM(a) method is employed, in which the resulting ratio spectra are directly utilized for calculating the quantities of CPX and CAF in the mixes. CPX content in the mixes was determined using regression equation: (P_235 nm_ – P_225 nm_) = 0.0774 C_CPX_ + 0.0048. Also, concentration of CAF in the mixes has been determined using regression equation: P constant = 0.0296 C_CAF_ + 0.0073. Alternatively, FRM(b) is employed to replicate D^0^ of CPX and CAF^19,42^. D^0^ spectrum of CAF in each mixture has been obtained by multiplying each individual constant value (CV) by pure CAF 10.0 μg/mL (divisor), Fig. 4b. CAF content was determined using the regression equation: A_272nm_ = 0.0499 C_CAF_ + 0.0119. The pure CPX has been acquired by subtracting previously determined pure CAF from matching D^0^ of laboratory-prepared combinations, Fig. 4c. CPX content in the mixes was determined using the regression equation: A_222nm_ = 0.041 C_CPX_ + 0.0155.

### Validation of the suggested methods

An evaluation was conducted on many parameters mentioned in the ICH recommendations to confirm the analytical accuracy of suggested methodologies^35^. The working linearity range has been determined by considering practical limits of absorbance according to Beer’s law^46^. The presence of acceptable linearity was demonstrated by the satisfactory recovery, the proximity of correlation coefficients to one, and the negligible intercept values. Table 1 provides a summary of the linearity ranges for each approach, along with their regression equations.

**Table 1:** Assay parameters and method validation for the determination of Caffeine and Chlorphenoxamine HCl pure samples by the proposed spectrophotometric methods.

Drug	CAF							CPX						
Parameter	AR	EAD	FZM	FDM_b_	FRM_b_	FDM_a_	FRM_a_	AR	EAD	FZM	FDM_b_	FRM_b_	FDM_a_	FRMa
Wavelength (nm)	D˚ at 272					D^1^at 287	P at (222&280)	D˚ at 222					D^1^at 231	ΔP at (225&235)
Calibration range^a^(µg/mL)	3–35							3–45						
Slope	0.0499					0.104	0.0975	0.041					0.0983	0.112
Intercept	0.0119					0.0674	0.0126	0.0155					0.0671	0.0642
Correlation coefficient	0.9996					0.9995	0.9996	0.9997					0.9999	0.9997
Mean^a^	99.81					101.12	100.43	100.43					98.87	100.5
RSD%	0.997					1.311	1.151	1.151					0.984	1.104
Repeatability^a, b^	0.986					1.18	0.93	0.93					1.12	1.23
Inter-day precision^a, b^	1.64					1.67	1.88	1.88					1.57	1.69

^a^Average of three experiments.

^b^Relative standard deviation of three concentrations of (5.0, 15.0, 30 μg/mL) for CAF and CPX.

Accuracy of provided procedures has been ensured by the adequate percentage of recoveries achieved when assessing each drug at four different concentration levels (5µg/mL, 15µg/mL, 25µg/mL, and 35µg/mL) within the established range of linearity. The suggested procedures were used to investigate triplicates of three different concentrations on same day as well as on three consecutive days to assess the repeatability and intermediate precision of the approaches, respectively. The concentrations of CAF and CPX used were 5.0 µg/mL, 10.0 µg/mL, and 20.0 µg/mL Table 1. Ability to measure specified components in mixtures with varying ratios was confirmed by obtaining acceptable results for % recoveries and RSD% values, as shown in Table 2.

**Table 2:** Analysis of laboratory prepared mixtures of Caffeine and Chlorphenoxamine HCl by the proposed spectrophotometric methods.

Conc in µg/mL CAF: CPX	AR		EAD		FZM		FDM_a_		FDM_b_		FRM_a_		FRM_b_	
	CAF	CPX	CAF	CPX	CAF	CPX	CAF	CPX	CAF	CPX	CAF	CPX	CAF	CPX
10:4^a, b^	100.54	99.85	100.14	101.33	100.41	100.42	100.04	100.42	99.98	99.69	100.52	101.23	100.65	98.83
20:10^a^	101.3	98.75	99.86	100.05	99.84	99.72	100.81	101.61	101.82	101.09	99.45	100.42	101.82	101.09
10:10^a^	100.04	99.62	101.02	101.43	101.05	99.82	99.87	100.91	100.05	98.65	99.51	101.21	100.05	98.65
15:30^a^	99.42	100.32	100.56	100.61	101.1	98.51	101.3	99.82	99.41	101.34	98.42	100.34	99.41	101.34
25:40^a^	99.86	100.04	99.73	98.91	100.6	101.14	100.1	98.73	98.07	99.87	99.26	98.86	98.07	99.87
Mean ± RSD%	100.23 ± 0.72	99.72 ± 0.6	100.3 ± 0.6	100.47 ± 1.04	100.6 ± 0.52	99.92 ± 0.97	100.42 ± 0.61	100.3 ± 1.1	99.87 ± 1.35	100.13 ± 1.1	99.43 ± 0.75	100.41 ± 0.96	99.43 ± 0.75	100.41 ± 0.96

^a^Average of three experiments. ^b^Ratio present in dosage form.

### Assaying of Allergex® caffeine tablets

Spectrophotometric approaches established allowed for efficient quantification of CAF and CPX in their mixed tablets without need for previous separation. Furthermore, the lack of interfering from tablet additives and effective implementation of the extraction process have been thoroughly confirmed by employing the conventional addition procedure. Table 3 shows the achievement of successful recoveries with low RSD% values.

**Table 3:** Quantitative estimation of Caffeine and Chlorphenoxamine HCl in Allergex® Caffeine Tablets and application of standard addition Technique.

Drug	Allergex® Caffeine Tablets							Standard Addition Technique								
	**Found% ** ^**a**^ ** ± SD%**							**Taken(µg/mL)**	**Added(µg/mL)**	**Recovery% ** ^**b**^						
	**AR**	**EAD**	**FZM**	**FDM**_**a**_	**FDM**_**b**_	**FRM**_**a**_	**FRM**_**b**_			**AR**	**EAD**	**FZM**	**FDM**_**a**_	**FDM**_**b**_	**FRM**_**a**_	**FRM**_**b**_
**CAF**	99.81 ± 1.103	100.51 ± 1.07	101.02 ± 0.67	98.72 ± 0.86	100.62 ± 1.12	100.1 ± 0.71	100.1 ± 0.71	10	5	98.7	100.1	101.33	100.52	100.05	99.48	98.73
									10	99.16	99.42	100.81	101.03	98.89	101.66	99.62
									20	100.05	101.2	101.47	98.72	99.11	100.94	101.85
								Mean ± SD%		99.3 ± 0.62	100.24 ± 0.93	100.24 ± 0.93	100.1 ± 1.21	99.35 ± 0.62	100.1 ± 1.213	100.07 ± 1.61
**CPX**	99.87 ± 0.92	101.2 ± 1.1	98.43 ± 0.84	101.06 ± 1.21	99.70 ± 1.22	99.41 ± 0.95	99.41 ± 0.95	4	3	101.47	99.54	100.71	101.2	98.55	99.22	101.31
									4	99.38	100.07	101.46	100.59	99.61	100.73	100.52
									8	99.11	100.96	100.53	101.82	100.08	100.98	100.17
								Mean ± SD%		99.98 ± 1.29	100.19 ± 0.718	100.19 ± 0.718	101.2 ± 0.62	99.41 ± 0.78	101.2 ± 0.615	100.67 ± 0.58

^a^Average of five determinations. ^b^Average of three determinations.

### Content uniformity testing

The proposed approaches, which involve simplifying the manipulation stages and reducing solvent and energy usage per sample analysis, are efficient, rapid, and environmentally friendly solutions for evaluating the content homogeneity of Allergex® Caffeine tablets. According to the USP criteria, dosage unit’s consistency was evaluated using the "content uniformity method" instead of the “weight variation” approach. This decision was made because the units that were evaluated were film-coated tablets with a drug substance ratio of less than 25%^43^. The manufacturing batch is considered accepted according to the requirements if the estimated acceptance value (AV) is less than 15, which corresponds to upper limit of allowable acceptance value (L1). Acceptance value (AV) has been determined by applying the following mathematical formula.:$AV\; = \;\left| M \right. - X^{ - } \left| { + K \times } \right.SD$

The proposed procedures have been utilized to analyze ten pills. Mean recovery percent (X^–^) of these tablets ranged from 98.50% to 101.50%, as shown in Table 4. In this situation, reference value (M) was equivalent to mean (X^–^), as well as acceptance value (AV) was simply result of multiplying acceptability constant (k) by the sample standard deviation (SD), with k being set at 2.4 when using a sample size of 10 (i.e. AV = 2.4 SD)^29^. Estimated average values (AV) have been observed to be smaller than L1, indicating a uniform distribution of drug content throughout the dose units, as shown in Table 4.

**Table 4:** Results of content uniformity testing for determining Caffeine and Chlorphenoxamine HCl in Allergex® Caffeine Tablets using the proposed spectrophotometric methods.

Allerge® Caffeine Tablet no	Label Claim (%)													
	CAF							CPX						
	AR	EAD	FZM	FDM_a_	FDM_b_	FRM_a_	FRM_b_	AR	EAD	FZM	FDM_a_	FDM_b_	FRM_a_	FRM_b_
1	101.36	100.52	99.75	100.72	98.22	98.85	100.31	99.58	100.13	100.56	101.4	100.42	99.76	101.83
2	100.08	101.21	101.04	101.61	99.76	100.82	101.56	100.05	99.57	101.45	100.03	99.87	100.52	100.52
3	101.17	97.98	100.54	99.64	100.54	101.24	99.82	100.79	98.73	100.06	99.1	98.21	100.83	99.27
4	99.83	99.76	101.26	99.15	101.73	101.06	100.77	101.39	101.79	99.87	98.93	99.54	101.08	98.17
5	100.75	101.12	100.31	100.52	99.44	99.73	98.84	100.67	97.96	98.54	97.96	100.57	99.75	100.45
6	101.43	98.85	99.56	100.97	100.87	98.52	101.11	99.56	99.59	99.78	101.32	101.02	100.67	99.42
7	98.74	100.65	98.54	98.84	100.08	100.91	99.34	98.84	101.09	100.42	100.61	100.5	101.06	98.98
8	97.85	100.34	99.12	100.09	98.84	100.46	100.33	100.81	100.34	101.31	101.54	98.84	100.41	101.32
9	100.52	101.56	101.42	101.69	100.42	99.43	98.42	101.31	101.4	99.69	100.36	99.51	99.85	100.7
10	99.08	98.91	100.6	100.07	101.21	98.57	100.51	100.07	100.18	100.72	101.04	100.77	100.52	98.22
Mean	100.08	100.09	100.21	100.33	100.111	99.96	100.1	100.31	100.08	100.24	100.23	99.23	100.45	99.88
SD	1.207	1.18	0.951	0.960	1.078	1.07	0.996	0.827	1.182	0.853	1.213	0.903	0.505	1.263
RSD %	1.206	1.18	0.949	0.957	1.076	1.071	0.995	0.825	1.182	0.851	1.211	0.904	0.503	1.264
AV ^a^	2.897	2.832	2.278	2.297	2.583	2.57	2.388	1.985	2.837	2.047	2.911	2.17	1.212	3.034

^a^Acceptance value = 2.4 × SD with maximum allowed level (L1) is 15.

### Statistical analysis

In order to assess the efficacy of suggested techniques to quantify the researched drugs, a statistical analysis was performed using the student t-test and F-test. This study aimed to conduct a comparative analysis among results attained from the proposed approaches with those acquired from the published HPLC method^8^. By establishing the p-value to 0.05, it was determined that the calculated Student t- and F-values were smaller than their respective tabular values. This indicates that there is no significant difference between the suggested methods and reported HPLC technique, Table 5.

**Table 5:** Statistical comparison of the results obtained by the proposed spectrophotometric methods and reported HPLC method for the analysis of Caffeine and Chlorphenoxamine HCl.

Items	CAF								CPX							
	**AR**	**EAD**	**FZM**	**FDM**_**b**_	**FRM**_**b**_	**FDM**_**a**_	**FRM**_**a**_	**Reported method ** ^**a**^	**AR**	**EAD**	**FZM**	**FDM**_**b**_	**FRM**_**b**_	**FDM**_**a**_	**FRM**_**a**_	**Reported method ** ^**a**^
Mean	99.81					100.08	100.52	99.43	100.86					100.77	100.5	100.06
SD	0.997					1.311	1.121	0.60	1.01					0.984	0.86	0.47
N	6								6							
Variance	0.99					1.72	1.26	0.36	1.02					0.97	0.74	0.22
Student’s t test^b^ (2.23)	0.801					1.104	2.09		1.76					1.594	1.1	
F value^b^ (5.05)	2.75					4.77	3.5		4.64					4.41	3.36	

^a^HPLC method with mobile phase consisting of ethyl acetate/methanol (50:50 v/v) /triethylamine pH 9 using Column: Shimpack NP-Sil. (150mm x 6mm i.d.,5μm), (Shimadzu, Japan)., isocratic elution, and UV detection at 254 nm. ^b^Values in parentheses are the corresponding theoretical values for t and F at P = 0.05.

### Greenness and whiteness assessment tools

Conformity of suggested methods to principles of green analytical chemistry has been evaluated using several assessment tools to provide a comprehensive understanding of the methods’ environmental friendliness^18,47–50^. In addition, they offered an extensive benchmark to evaluate their environmental impact in comparison to any future established method for CAF and CPX mixture. The evaluation has been carried out using multi-tools (NEMI Pictogram, Analytical Eco- Scale, GAPI Tool, AGREE Tool, and RGB Model), Table 6. Further details regarding the greenness can be found in the supplementary data.

**Table 6:** Greenness assessment of Spectrophotometric methods and reported HPLC method via different metrics.

Analytical Eco-scale		Complex GAPI Tool	AGREE Tool	RGB Model
**Spectrophotometric Method**				
Reagent				
Water	0			
Instruments				
Energy	0			
Occupational Hazard	0			
Waste	6			
Total Penalty points	6			
Total score	94			
**Reported HPLC Method**				
Reagent				
Methanol	6			
Acetonitrile	6			
Triethylamine	8			
Instruments				
Energy	1			
Occupational Hazard	0			
Waste	6			
Total Penalty points	27			
Total score	73			

## Conclusion

Suggested approaches are characterized by their simplicity, speed, affordability, as well as high level of selectivity. Recently introduced methods allowed for the accurate and precise measurement of CAF and CPX, in their pure forms and in pharmaceutical dosage forms. Analysis has been unaffected by common excipients, as evidenced by the obtained results. Using of diverse factorized response spectra renders these methods appropriate for the resolution of a broad range of analyte mixtures that possess overlapping spectra, whether partially or entirely. The chosen methodology employed water-based spectrophotometry instead of traditional chromatography due to its superior attributes, including exceptional environmentally friendly practices of water, reduced usage of solvents and energy, as well as the lack of dependence on highly skilled analysts. Furthermore, this approach is simpler, faster, and more cost-effective. The proposed techniques were effectively utilized not only to assess the potency of the produced tablets but also for ensuring their uniformity of content. Additionally, sustainability of the proposed methods has been evaluated using Analytical Eco-Scale, complex GAPI pictograms and the AGREE tool, which confirmed their advantage compared to the stated HPLC. Ultimately, a comprehensive analysis of the proposed approaches has been conducted using the WAC tool, highlighting degree to which the methods align with WAC principles.

## Supplementary Information

Supplementary Information.

## Data Availability

The authors declare that the data supporting the findings of this study are available within the paper and its Supplementary Information files. Should any raw data files be needed in another format they are available from the corresponding author upon reasonable request.

## References

[1] Motola, D. et al. Safety profile of H1-antihistamines in pediatrics: An analysis based on data from VigiBase. *Pharmacoepidemiol. Drug Saf.* **26**, 1164–1171. 10.1002/pds.4246 (2017). 28653802 10.1002/pds.4246

[2] Chlorphenoxamine monograph. The United States Pharmacopeia 20. The National Formulary. Rockville, Md (Pharmacopeial convention, United States, Inc., 1979)

[3] Schafer, A. et al. Repurposing potential of 1st generation H1-specific antihistamines as anti-filovirus therapeutics. *Antiviral Res.* **157**(2018), 47–56. 10.1007/s40265-017-0830-1(accessedMarch5 (2024). 10.1016/j.antiviral.2018.07.003 PMC6087678 29981374

[4] Travi, B. L. Current status of antihistamine drugs repurposing for infectious diseases. *Med. Drug Discov.* **15**, 2024. 10.1007/s40265-017-0830-1(accessedMarch5 (2022).

[5] Dyall, J. et al. Middle east respiratory syndrome and severe acute respiratory syndrome: Current therapeutic options and potential targets for novel therapies. *Drugs* **77**(2017), 1935–1966. 10.1007/s40265-017-0830-1(accessedMarch5 (2024). 10.1007/s40265-017-0830-1 PMC5733787 29143192

[6] Caffeine monograph. The United States Pharmacopeia 35. The National Formulary. Rockville, Md.; United States Pharmacopeial convention. https://www.drugfuture.com/Pharmacopoeia/usp35/PDF/2427-2428%20Caffeine.pdf.

[7] Fredholm, B. B., Bättig, K., Holmén, J., Nehlig, A. & Zvartau, E. E. Actions of caffeine in the brain with special reference to factors that contribute to its widespread use. *Pharmacol. Rev.* **51**, 83–133 (1999). 10049999

[8] Dessouky, Y. M., Hassanein, H. H., Mohammad, A.-A. & Hanafy, R. S. Normal phase high performance liquid chromatographic determination of chlorphenoxamine hydrochloride, caffeine and 8-chlorotheophylline. *Bull. Fac. Pharm. Cairo. Univ.* **42**(1), 53–63 (2004).

[9] Ashraf, A., ElDin, N. B., Rostom, Y., El-Zeany, B. A. & Sedik, G. A. Novel RP-HPLC–DAD approach for simultaneous determination of chlorphenoxamine hydrochloride and caffeine with their related substances. *BMC Chem.* **18**, 1–11. 10.1186/s13065-024-01238-8 (2024). 39030644 10.1186/s13065-024-01238-8 PMC11264915

[10] Bebawy, L. I. & El-Kousy, N. M. Simultaneous determination of some multicomponent dosage forms by quantitative thin layer chromatography densitometric method. *J. Pharm. Biomed. Anal.*10.1016/S0731-7085(99)00039-4 (1999). 10704135 10.1016/s0731-7085(99)00039-4

[11] H.M. Lotfy, H. Salem, A.M. Mohsen, H.M. Lotfy, A.M. Badawey, S.Z. Elkhateeb, Application of three novel spectrophotometric methods manipulating ratio spectra for resolving a pharmaceutical mixture of Chlorphenoxamine hydrochloride and Caffeine, (2013).

[12] Dinç, E., Palabıyık, İM., Üstündağ, Ö., Yurtsever, F. & Onur, F. Simultaneous spectrophotometric determination of chlorphenoxamine hydrochloride and caffeine in a pharmaceutical preparation using first derivative of the ratio spectra and chemometric methods. *J. Pharm. Biomed. Anal.* **28**, 591–600. 10.1016/S0731-7085(01)00694-X (2001). 10.1016/s0731-7085(01)00694-x 12008138

[13] Kelani, K. M. Simultaneous determination of caffeine, 8-chlorotheophylline, and chlorphenoxamine hydrochloride in ternary mixtures by ratio-spectra zero-crossing first-derivative spectrophotometric and chemometric methods. *J. AOAC Int.*10.1093/jaoac/88.4.1126 (2005). 16152931

[14] Abdel-Ghani, N. T., Abu-Elenien, G. M. & Hussein, S. H. Differential pulse voltammetric determination of chlorphenoxamine hydrochloride and its pharmaceutical preparations using platinum and glassy carbon electrodes. *J. Appl. Electrochem.* **40**(2010), 499–505. 10.1007/s10800-009-0021-1(accessedMarch5 (2024).

[15] Abdel-Ghani, N. T. & Hussein, S. H. Flow injection potentiometric determination of Chlorphenoxamine Hydrochloride. *J. Appl. Electrochem.* **40**(2010), 2077–2090. 10.1007/s10800-010-0189-4(accessedMarch5 (2024).

[16] Ashraf, A., El Zeany, B. A., Sedik, G., Rostom, Y. & Ibrahim, N. Novel green screen-printed potentiometric sensor for monitoring antihistamine drug chlorphenoxamine HCl in various matrices. *J. Electrochem. Soc.*10.1149/1945-7111/ad659e (2024).

[17] Rezk, M. R., Tantawy, M. A., Wadie, M. & Weshahy, S. A. Smart spectrophotometric assessment of tamsulosin hydrochloride and tadalafil in their new pharmaceutical formulation for treatment of benign prostatic hyperplasia and erectile dysfunction. *Spectrochim. Acta A Mol. Biomol. Spectrosc.* **227**, 117547. 10.1016/j.saa.2019.117547 (2020). 31734571 10.1016/j.saa.2019.117547

[18] Rostom, Y., Wadie, M., Rezk, M. R., Marzouk, H. M. & Abdel-Moety, E. M. Fingerprinting and iso-absorptive resolution techniques for the spectrally overlapping Dutasteride and Silodosin mixture: Content uniformity testing along with greenness profile assessment. *Spectrochim. Acta A Mol. Biomol. Spectrosc.* **273**, 121063. 10.1016/j.saa.2022.121063 (2022). 35219273 10.1016/j.saa.2022.121063

[19] Hegazy, M. A., Lotfy, H. M., Rezk, M. R. & Omran, Y. R. Novel spectrophotometric determination of chloramphenicol and dexamethasone in the presence of non-labeled interfering substances using univariate methods and multivariate regression model updating. *Spectrochim. Acta A Mol. Biomol. Spectrosc.* **140**, 600–613. 10.1016/j.saa.2014.12.098 (2015). 25659506 10.1016/j.saa.2014.12.098

[20] Lotfy, H. M., Hegazy, M. A., Rezk, M. R. & Omran, Y. R. Comparative study of novel versus conventional two-wavelength spectrophotometric methods for analysis of spectrally overlapping binary mixture. *Spectrochim. Acta A Mol. Biomol. Spectrosc.* **148**, 328–337. 10.1016/j.saa.2015.04.004 (2015). 25909908 10.1016/j.saa.2015.04.004

[21] Rezk, M. R., Monir, H. H. & Marzouk, H. M. Spectrophotometric assessment of the brand-new antiviral combination: Sofosbuvir and velpatasvir in their pure forms and pharmaceutical formulation. *Spectrochim. Acta A Mol. Biomol. Spectrosc.* **213**, 159–166. 10.1016/j.saa.2019.01.058 (2019). 30685554 10.1016/j.saa.2019.01.058

[22] Lotfy, H. M., Obaydo, R. H. & Mohamed, E. H. environmentally sustainable computationally spectrophotometric resolution strategy for analysis single-tablet regimens of antihypertension with overlapped spectra. *Talanta Open* **7**, 100226. 10.1016/j.saa.2019.01.058 (2023).

[23] Rezk, M. R., Abdel-Moety, E. M., Wadie, M. & Tantawy, M. A. Stability assessment of tamsulosin and tadalafil co-formulated in capsules by two validated chromatographic methods. *J. Sep. Sci.* **44**, 530–538. 10.1002/jssc.202000975 (2021). 33207075 10.1002/jssc.202000975

[24] Rezk, M. R., Basalious, E. B. & Badr, K. A. Novel determination of sofosbuvir and velpatasvir in human plasma by UPLC–MS/MS method: Application to a bioequivalence study. *Biomedical. Chromatogr.* **32**, e4347. 10.1002/bmc.4347 (2018). 10.1002/bmc.4347 30047564

[25] Hussein, O. G., Rostom, Y., Abdelkawy, M., Rezk, M. R. & Ahmed, D. A. Spectrophotometric platform windows’ exploitation for the green determination of Alcaftadine in presence of its oxidative degradation product. *Spectrochim. Acta A Mol. Biomol. Spectrosc.*10.1016/j.saa.2023.122737 (2023). 37075686 10.1016/j.saa.2023.122737

[26] Gałuszka, A., Migaszewski, Z. & Namieśnik, J. The 12 principles of green analytical chemistry and the SIGNIFICANCE mnemonic of green analytical practices. *TrAC Trends in Anal. Chem.* **50**, 78–84. 10.1016/j.trac.2013.04.010 (2013).

[27] Kurowska-Susdorf, A. et al. Green analytical chemistry: Social dimension and teaching. *TrAC Trends Anal. Chem.* **111**, 185–196. 10.1016/j.trac.2018.10.022 (2019).

[28] Płotka-Wasylka, J., Fabjanowicz, M., Kalinowska, K. & Namieśnik, J. History and milestones of green analytical chemistry. in *Green Analytical Chemistry: Past, Present and Perspectives*. 1–17. http://www.springer.com/series/11661 (2019).

[29] Stage 6 Harmonization Official, (2011).

[30] Gałuszka, A., Migaszewski, Z. M., Konieczka, P. & Namieśnik, J. Analytical Eco-Scale for assessing the greenness of analytical procedures. *TrAC - Trends Anal. Chem.* **37**(2012), 61–72. 10.1016/j.trac.2012.03.013(accessedMarch6 (2024).

[31] Płotka-Wasylka, J. A new tool for the evaluation of the analytical procedure: Green analytical procedure index. *Talanta* **181**, 204–209. 10.1016/j.talanta.2018.01.013 (2018). 29426502 10.1016/j.talanta.2018.01.013

[32] Płotka-Wasylka, J. & Wojnowski, W. Complementary green analytical procedure index (ComplexGAPI) and software. *Green Chem.* **23**, 8657–8665. 10.1039/D1GC02318G (2021).

[33] Pena-Pereira, F., Wojnowski, W. & Tobiszewski, M. AGREE - Analytical greenness metric approach and software. *Anal. Chem.* **92**(2020), 10076–10082. 10.1021/acs.analchem.0c01887(accessedMarch6 (2024). 10.1021/acs.analchem.0c01887 PMC7588019 32538619

[34] Nowak, P. M., Wietecha-Posłuszny, R. & Pawliszyn, J. White analytical chemistry: An approach to reconcile the principles of green analytical chemistry and functionality. *TrAC - Trends Anal. Chem.*10.1016/j.trac.2021.116223 (2021).

[35] ICH guidelines (Q2R1), ICH Harmonised Tripartite Guideline Validation of Analytical Procedure: Text and Methodolgy Q2(R1) Retrieved from the International Conference on Harmonisation of Technical Requirements for Registration of Pharmaceuticals for Human Use www.somatek.com (2014)

[36] Lotfy, H. M., Elshahed, M. S. & Mohamed, D. The concept of relative absorptivity distribution for enhancing disbanding power of spectrophotometric technique to resolve co-formulated tablets: A tool for purity index and uniformity assessment. *Spectrochim. Acta A Mol. Biomol. Spectrosc.*10.1016/j.saa.2020.118551 (2020). 32502814 10.1016/j.saa.2020.118551

[37] Morgan, E. M., Boltia, S. A., Fayez, Y. M., Abdelkawy, M. & Lotfy, H. M. Coupling of physical extraction and mathematical filtration in spectrophotometric analysis of natural therapy essential for prophylaxis and treatment of COVID-19 infection - Comparative study along with greenness evaluation. *Heliyon*10.1016/j.heliyon.2023.e16284 (2023). 37235204 10.1016/j.heliyon.2023.e16284 PMC10193772

[38] El-Hanboushy, S., Marzouk, H. M., Fayez, Y. M., Abdelkawy, M. & Lotfy, H. M. Sustainable spectrophotometric determination of antihypertensive medicines reducing COVID-19 risk via paired wavelength data processing technique - Assessment of purity, greenness and whiteness. *Sustain. Chem. Pharm.*10.1016/j.scp.2022.100806 (2022). 35992213 10.1016/j.scp.2022.100806 PMC9376343

[39] Ahmed, D. A. & Lotfy, H. M. Sticking-pulling strategy for assessment of combined medicine for management of tough symptoms in COVID-19 Pandemic using different windows of spectrophotometric Platform-Counterfeit products’ detection. *Spectrochim. Acta A Mol. Biomol. Spectrosc.* **277**, 121256. 10.1016/j.saa.2022.121256 (2022). 35483258 10.1016/j.saa.2022.121256 PMC9759764

[40] Lotfy, H. M., Mohamed, D. & Elshahed, M. S. Novel univariate spectrophotometric determination of the recently released solid dosage form comprising dapagliflozin and saxagliptin via factorized response spectra: Assessment of the average content and dosage unit uniformity of tablets. *Spectrochim. Acta A Mol. Biomol. Spectrosc.* **222**, 117120. 10.1016/j.saa.2019.05.025 (2019). 31252262 10.1016/j.saa.2019.05.025

[41] Lotfy, H. M. & Omran, Y. R. Novel absorptivity centering method utilizing normalized and factorized spectra for analysis of mixtures with overlapping spectra in different matrices using built-in spectrophotometer software. *Spectrochim. Acta A Mol. Biomol. Spectrosc.* **200**, 167–178. 10.1016/j.saa.2018.04.004 (2018). 29680495 10.1016/j.saa.2018.04.004

[42] Ahmed, D. A. & Lotfy, H. M. Evaluation of in silico and in lab sample enrichment techniques for the assessment of challengeable quaternary combination in critical ratio. *Spectrochim. Acta A Mol. Biomol. Spectrosc.* **260**, 119943. 10.1016/j.saa.2021.119943 (2021). 34030038 10.1016/j.saa.2021.119943

[43] Hudson-Curtis, B. & Novick, S. Assessing content uniformity. In *Nonclinical Statistics for Pharmaceutical and Biotechnology Industries* (ed. Zhang, L.) 631–651 (Springer International Publishing, 2016).

[44] Madhuri, R. Water as the Green Solvent in Organic Synthesis. In *Industrial Applications of Green Solvents - II* 182–201 (Materials Research Forum LLC, 2019).

[45] Lotfy, H. M., Monir, H. H., Erk, N. & Rostom, Y. Novel feature extraction approach for achieving potential spectral resolution: Green analytical application on zofenopril calcium and hydrochlorothiazide in their spectrally overlapping binary mixture. *Spectrochim. Acta A Mol. Biomol. Spectrosc.* **230**, 117998. 10.1016/j.saa.2019.117998 (2020). 31931351 10.1016/j.saa.2019.117998

[46] Rostom, Y. et al. Trade-off efficacy and data processing strategy in the power of spectral resolution of co-formulated antihypertensive pharmaceuticals. *Spectrochim. Acta A Mol. Biomol. Spectrosc.* **247**, 119080. 10.1016/j.saa.2020.119080 (2021). 33126135 10.1016/j.saa.2020.119080

[47] Carraro, C. & Siniscalco, D. Strategies for the international protection of the environment. *J. Public Econ.* **52**, 309–328. 10.1016/0047-2727(93)90037-T (1993).

[48] Capello, C., Fischer, U. & Hungerbühler, K. What is a green solvent? A comprehensive framework for the environmental assessment of solvents. *Green Chem.* **9**, 927–993. 10.1039/B617536H (2007).

[49] Keith, L. H., Gron, L. U. & Young, J. L. Green analytical methodologies. *Chem. Rev.* **107**(2007), 2695–2708. 10.1021/cr068359e(accessedMarch6 (2024). 10.1021/cr068359e 17521200

[50] Soliman, R. M. et al. Novel fabricated potentiometric sensors for selective determination of carbinoxamine with different greenness evaluation perspectives. *Microchemical. J.*10.1016/j.microc.2022.10838 (2023).

