# Supplementary material for: Smart green spectrophotometric estimation and content uniformity testing of chlorphenoxamine HCl and caffeine in bulk forms and combined pharmaceutical formulation
Source: Sci Rep. 2025 Mar 19;15:9482. doi: 10.1038/s41598-025-92166-7 (PMC11923055; doi:10.1038/s41598-025-92166-7)
Supplement: Supplementary file 1 — Supplementary Information. [file 41598_2025_92166_MOESM1_ESM.docx]

**Smart green spectrophotometric estimation and content uniformity testing of chlorphenoxamine HCl and caffeine
in bulk forms and combined pharmaceutical formulation**

Ahmed Ashraf*, Ghada A. Sedik, Badr A. El-Zeany, Yasmin Rostom

Analytical Chemistry Department, Faculty of Pharmacy, Cairo University, Kasr El-Aini Street, 11562 Cairo, Egypt
* Corresponding Author: ahmed.ashraf@pharma.cu.edu.eg

**3.8. Greenness and Whiteness Assessment Tools**

Conformity of suggested methods to principles of green analytical chemistry was evaluated using several assessment tools to provide a comprehensive understanding of the methods' environmental friendliness[18,48–51]. In addition, they offered an extensive benchmark to evaluate their environmental impact in comparison to any future established method for CAF and CPX mixture.

**3.8.1. Analytical Eco-Scale System**

In order to assess the environmental impact of the analytical process, a new grading system has been implemented that assigns penalty points [30].. Every element of the analytical procedure that affects the quality of the analysis, such as the amount of chemical used, waste generated, or energy consumed, is given a penalty point, which is deducted from the base value of 100 for the perfect green technique. Table 6 displays the Eco-Scale rating. The method's efficacy is validated by a score exceeding 75. Table 6 displays an illustration of the penalty points allocated to the established spectrophotometric method and the documented HPLC technique. The current method demonstrates an excellent Eco-score, whereas the aforementioned HPLC process only achieves a moderately satisfactory score due to its significant reliance on hazardous organic solvents.

**3.8.2. Complimentary green analytical procedure index (Complex GAPI)**

Given that the choice of solvents is not the sole determinant of the environmental friendliness of an analytical method, three cutting-edge criteria were employed to comprehensively assess all aspects of the method's life cycle. The initial measure that was implemented was Complex GAPI, which is an enhanced version of the widely-used GAPI tool developed by Justyna in 2018 [52]. This tool has been utilized by numerous researchers to evaluate their suggested approaches. A protocol consisting of fifteen parameters was adhered to in order to conduct a thorough and partially quantitative assessment of the entire analytical processes, beginning with sample preparation, transit, storage, solvents, and concluding with instrumental analysis. A trichromatic pictogram was utilized to provide rapid and effortless visual assessment and comparison. Following that, Complex GAPI was introduced as a user-friendly software solution designed to simplify complex tasks. It also takes into account the potential negative impact of any preliminary analysis based on eleven specific parameters [32]. The expansion was indicated by the presence of an extra hexagonal area located in the lower part of the GAPI pictogram. When comparing our suggested UV-methods with the mentioned HPLC, it was seen that the UV-methods exhibited a greater prevalence of green color. This may be attributed to the fact that water has a higher greenness characteristic in addition to the lower waste and energy consumption per sample analysis, table 6. It is important to note that additional hexagonal has not been assigned a color due to the lack of pre-analytical methods for sample analysis.

**3.8.3. Analytical greenness (AGREE) metric**

In addition to Complex GAPI, evaluation of techniques sustainability was also assessed using the recently available AGREE program [33], which provided a more quantitative approach. Conformity of the analytical procedure to each principle of GAC was assessed and represented by a circular pictogram consisting of twelve sections, with colors ranging from deep green to full red. Ultimately, a comprehensive assessment was conducted, resulting in the assignment of a score ranging from zero to one. A higher score signifies a greater level of sustainability, Figure S-1. According to table 6, spectrophotometric approaches achieved a higher overall score (0.92) compared to the published HPLC method (0.66).

**3.8.4. White analytical chemistry assessment (RGB12 algorithm tool)**

From an analytical chemistry perspective, it is important to note that not all methods that are labeled as "green" are necessarily sustainable. This is because green methods primarily focus on minimizing harm to the environment but may not address other aspects of sustainability. Sustainability encompasses aspects beyond environmental friendliness, including the effectiveness, validity, and affordability of an approach [34]. Hence, sustainable techniques aim to achieve a comprehensive equilibrium among the sustainability, performance, and productivity of analytical procedures, while also revealing imperfections. The RGB12 algorithm tool was built to ensure the sustainability of the approaches. This tool incorporates a freely accessible resource, table 6. Evaluation of the greenness profile of the proposed spectrophotometric method, the reported HPLC method, and the Complex GAPI, AGREE, and white assessment tools. The composition consists of tables in red, green, and blue, which encompass the twelve WAC assumptions. The red table specifically addresses analytical performance in relation to sensitivity, precision, and accuracy. The green table assesses the sustainability of the process in terms of amount of chemical, reagent toxicity, and other hazards, while the blue table offers information on cost, time, and sample material consumption. The final summary is offered in the form of a table consisting of three columns colored in red, green, and blue. This table provides a straightforward representation of the level of adherence to WAC postulates, with a numerical score out of 100 displayed at the bottom. The most effective and lasting strategy would be the one that has the highest percentages of three shades, which in turn contribute to the highest whiteness score. In this study, the RGB12 algorithm tool shown comparable analytical performance to the reported HPLC approach for analyzing the marketed dosage forms, Figure S-2. However, the UV method had a broader range of applications due to its capacity to verify content uniformity. Despite this, the proposed UV methods outperformed in terms of sustainability and functionality due to the environmentally friendly nature of water, minimal energy usage, and ease of application.


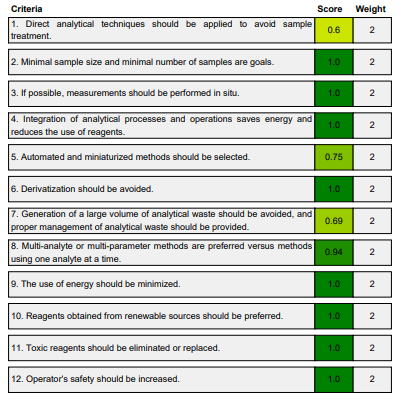


**Figure S-1: The twelve principles of AGREE**


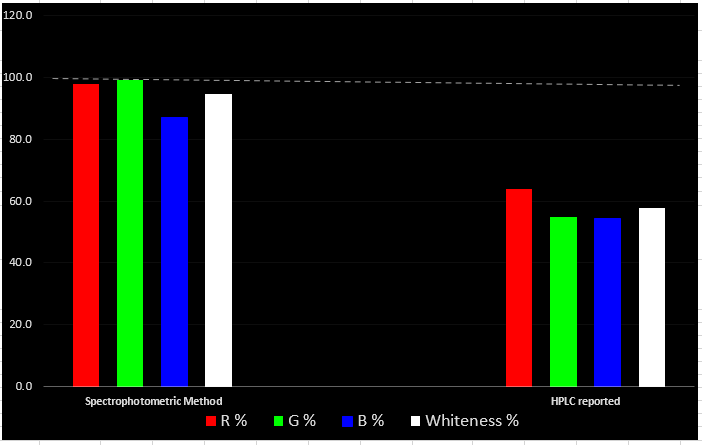


**Figure S-2: Comparison of whiteness assessment between spectrophotometric method and reported HPLC method**
